# Analysis of Adolescent HIV Care Cascade Outcomes in PEPFAR-Supported Programs in Central America, October 2020–September 2024

**DOI:** 10.3390/tropicalmed11010005

**Published:** 2025-12-24

**Authors:** Lissette Raquel Chang, Cristine Gutierrez, Jose Rodas, Nancy Aitcheson, Nasim Farach, Carlos Castaneda, Andres Azmitia Rugg, Benjamin Ryan Phelps

**Affiliations:** 1 Centers for Disease Control and Prevention (CDC), Division of Global HIV & TB, Panama City 0801, Panama; 2 Centers for Disease Control and Prevention (CDC), Division of Global HIV & TB, Guatemala City 01016, Guatemalaao73@cdc.gov (B.R.P.); 3 Centers for Disease Control and Prevention (CDC), Division of Global HIV & TB, Atlanta, GA 30333, USA; 4 Centers for Disease Control and Prevention (CDC), Division of Global HIV & TB, Tegucigalpa 11101, Honduras; 5 Centers for Disease Control and Prevention (CDC), Division of Global HIV & TB, San Salvador 1101, El Salvador

**Keywords:** adolescent, HIV, treatment, advanced HIV disease, viral load suppression

## Abstract

To better understand recent adolescent (10–19 years) HIV trends in Central America, we analyzed routine data from countries supported by the United States President’s Emergency Plan for AIDS Relief (PEPFAR): Guatemala, El Salvador, Honduras, Panama, and Nicaragua, over the period from October 2020 to September 2024. Key PEPFAR indicators included HIV testing, HIV positivity rates, new treatment initiations, advanced HIV disease (AHD) at diagnosis, viral load coverage (VLC), viral load suppression (VLS), and multi-month dispensing (MMD) uptake for children and adolescents living with HIV (CALHIV) from 10–19 years of age. Since October 2020, the number of HIV tests conducted among adolescents has increased; however, the positivity rate has remained stable at approximately 2%. The number of adolescents initiating treatment increased by 21%. At the same time, VLS has shown steady regional improvement (from 73% to 90%), though VLC is a persistent challenge (80%). Treatment interruption rates have been relatively stable, fluctuating between 2% and 3%. Advanced HIV is high in adolescents new to treatment (34%), especially among females (40%), though cluster of differentiation 4 (CD4) testing at diagnosis has only been collected recently and coverage is not complete. The high prevalence of AHD among adolescents underscores the need to reinforce earlier and more targeted interventions for adolescents, especially in countries with greater HIV prevalence such as Panama and Guatemala.

## 1. Introduction

Despite progress in reducing the global burden of HIV, adolescents living with HIV (ALHIV) remain an overlooked population, with persistent gaps across the HIV care continuum. They experience higher rates of delayed diagnosisand poorer outcomes in retention and viral suppression compared to adults [1].

International guidelines emphasize the need for adolescent-responsive services to address transitions into adulthood, HIV status disclosure, and differentiated services appropriate for adolescents [1,2] Additionally, many programs do not consider challenges beyond HIV, such as the prevention of pregnancies, the prevention of sexually transmitted infections, substance abuse, violence, and the need to establish life goals [3,4,5].

According to 2024 global data from the Joint United Nations Programme on HIV/AIDS (UNAIDS), the HIV epidemic in Central America is heterogeneous. Guatemala has the largest estimated number of people living with HIV (PLHIV), with 33,000 individuals and a prevalence of 0.2%. Panama, while having a slightly smaller number of PLHIV (31,000), demonstrates the highest prevalence at 1.1%. El Salvador follows with 21,000 PLHIV and a prevalence of 0.4%, followed by Honduras with 19,000 PLHIV and a prevalence of 0.2%. These patterns suggest a concentrated epidemic in specific networks and geographies [6,7,8,9,10] During this same period, adolescents (10–19 years) accounted for 4% of PLHIV globally (1.6 million), of which about 4000 were in Central America. Recent reports from UNAIDS and HIV researchers have described progress with regards to the HIV response in this region; however, gaps remain in testing and treatment coverage [11,12,13,14].

**Figure 1:**
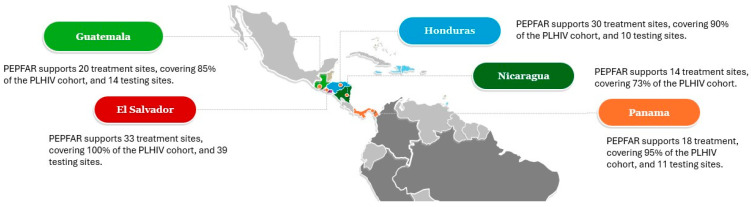
Number of PEPFAR-supported sites across Central American in 2024. Created by the authors using data from DATIM (Data for Accountability, Transparency, and Impact).

The U.S. President’s Emergency Plan for AIDS Relief (PEPFAR) supports five countries in Central America: Guatemala, El Salvador, Honduras, Panama, and Nicaragua, reaching 70,983 people living with HIV in 2024 (89% coverage of all PLHIV in this region), of whom approximately 2% were adolescents aged 10–19 years (Figure 1). Most care and treatment sites in Guatemala, El Salvador, and Panama receive some support through PEPFAR programming, whereas in Honduras and Nicaragua, most of these sites are supported by the Ministries of Health. In contrast, PEPFAR does not offer support to the majority of national HIV testing sites across the five countries. PEPFAR plays a crucial role in addressing the HIV/AIDS epidemic, collaborating with Ministries of Health and implementing a comprehensive approach through partners. This approach includes HIV prevention, testing, treatment, the capacity building of HIV services, and the monitoring of key indicators.

Our analysis evaluates recent trends in HIV testing, treatment initiation, and viral load suppression among adolescents aged 10–19 years in Central America from 2020 to 2024.

## 2. Materials and Methods

We conducted a retrospective, descriptive analysis of secondary data using routinely collected PEPFAR program data [15] from Guatemala, El Salvador, Honduras, Nicaragua, and Panama, covering the period from October 2020 to September 2024. This analysis included adolescents aged 10–19 years and used PEPFAR fiscal years, which run from October 1 to September 30 of the following year. Accordingly, the fiscal years were categorized as follows: October 2020–September 2021 (Year 1), October 2021–September 2022 (Year 2), October 2022–September 2023 (Year 3), and October 2023–September 2024 (Year 4).

PEPFAR program data were extracted from the DATIM (Data for Accountability, Transparency, and Impact) database [16], the official PEPFAR reporting system for the MER (Monitoring, Evaluation, and Reporting) indicators. DATIM contains de-identified, aggregate MER data reported by implementing partners from the sites supported, in whole or in part, by PEPFAR.

We conducted a descriptive year-on-year analysis of aggregate indicators and did not perform individual-level analyses, as DATIM does not include patient-level identifiers or longitudinal follow-up time. To compare trends across the years, between genders, across age groups, advanced stage diagnosis, and countries, χ^2^ and two-sample proportion tests were employed, with a cutoff of α = 0.05 for significance. Missing data was omitted from the analyses; given the aggregate nature of the data source, imputation was not possible. PEPFAR supports data collection, cleaning, and validation across supported countries; aggregate results are reported quarterly or semi-annually, according to PEPFAR MER guidelines.

### Indicators Used for Analysis

Key PEPFAR MER indicators included HIV testing (HTS_TST), HIV positive tests (HTS_TST_POS), new treatment initiations (TX_NEW), viral load coverage (VLC), viral load suppression (VLS), and multi-month dispensing (MMD) uptake [17] The definitions and additional indicators may be seen in Table 1. Indicators were selected in alignment with the conceptual framework of the HIV care cascade [18] and the UNAIDS 95-95-95 targets [19].

These indicators were reported for the entire cohort supported by PEPFAR funding in each country. For the purpose of this analysis, we limited results to indicators for which age disaggregation for ages 10–19 years was available. This analysis was reviewed by the U.S. Centers for Disease Control and Prevention and determined not to constitute human subject research.

To describe treatment continuity, we used interruption in treatment and MMD (months of ART dispensed) as programmatic proxies, recognizing that these do not constitute formal cohort retention estimates.

For this analysis we derived Advanced HIV Disease (AHD) among new ART initiators using the cluster of differentiation 4 (CD4) results reported in DATIM. Adolescents were classified as having AHD if their most recent CD4 result at or within three months of ART initiation was <200 cells/mm^3^ or if they were recorded as WHO stage 3 or 4 at initiation, consistent with WHO/UNAIDS definitions.

## 3. Results

### 3.1. HIV Testing

Since Year 1, the total number of HIV tests supported among adolescents increased across most of Central America, from 11,415 to 17,237 (Table 2). Regionally, 90% of adolescent HIV tests were performed in adolescents aged 15–19. El Salvador and Guatemala have consistently increased the number of tests conducted in adolescents from 2020 to 2024, whereas Honduras, Nicaragua, and Panama showed fluctuating patterns in testing volumes. An increase and subsequent decrease in testing were reported in Nicaragua each year of the analysis among those aged 15 to 19 years.

During Year 4, adolescents represented approximately 3.1% of all tested individuals in the region. El Salvador recorded the highest number of adolescent tests, totaling 5251, with all new adolescent diagnoses (32) in El Salvador occurring among those aged 15–19. Additional testing data can be found in Supplemental Table S1.

#### 3.1.1. Testing Modalities

**Figure 2:**
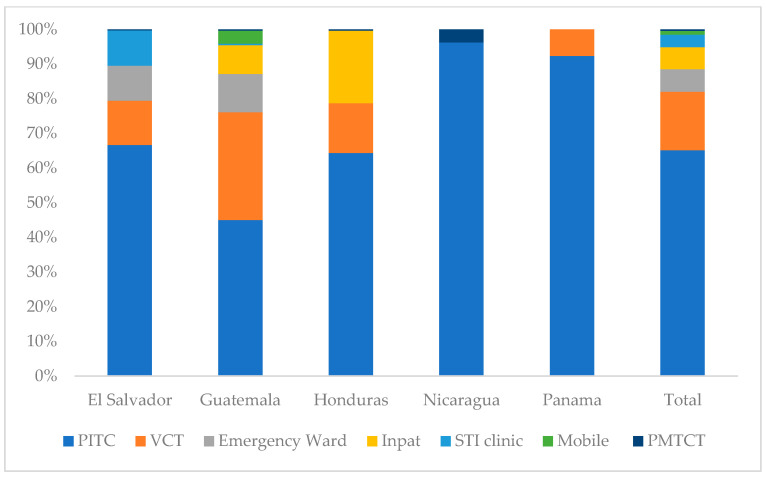
Test modality distribution by country, October 2023 to September 2024. Testing modalities: Emergency Ward = HIV testing in hospital emergency services; Inpat = HIV testing in inpatient hospital wards; PITC = provider-initiated testing and counseling during routine clinical visits; Mobile = community/mobile outreach testing services; PMTCT = testing in prevention of mother-to-child transmission services; STI clinic = testing in sexually transmitted infection clinics; VCT = voluntary counseling and testing services.

Across all countries, most HIV testing was conducted through facility-based, provider-initiated approaches, with varying contributions from voluntary and community-based modalities (Figure 2).

- In El Salvador 45% of tests were conducted through routine provider-initiated testing in health facilities (Other PITC), 22% through voluntary counseling and testing (VCT), and 11% in sexually transmitted infection (STI) clinics.
- In Guatemala, around 34% of HIV tests were conducted through routine health services in clinics and hospitals, 31% through voluntary testing services, and 11% through mobile outreach services.
- In Honduras, 54% of all HIV tests were conducted through routine health services, 24% through voluntary testing services, and 13% among inpatient clients.
- In Nicaragua 49% of all HIV tests were conducted in routine health services, 29% through voluntary testing services, and 10% through community-based outreach.
- Panama’s large majority of HIV tests were conducted through routine health services (71%), whereas only 11% were provided through voluntary testing services.

#### 3.1.2. HIV Positive Testing

Between Year 1 and Year 4, a total of 56,662 adolescents were tested: 1243 tested positive, yielding a regional positivity of 2.0%. Ninety-six percent of diagnoses occurred among adolescents between 15 and 19 years of age. Guatemala (3.1%) and Panama (4%) were the only countries reporting positivity rates above the regional average and required the fewest tests to identify one positive case, indicating higher yields compared with neighboring countries. By sex, adolescent males had a positivity proportion twice that of females (Table 2). When age was also considered, positivity was higher in both male age groups, 10–14 and 15–19 (1.6% and 2.9%, respectively), compared with females in same age groups (0.2% and 1.0%, respectively); however, these differences were not statistically significant (Supplemental Table S2). The highest positivity among males aged 15–19 was observed in Panama (4.9%).

#### 3.1.3. Country-Level Trends in Year 4

Country-level analysis revealed distinct patterns.

**Figure 3:**
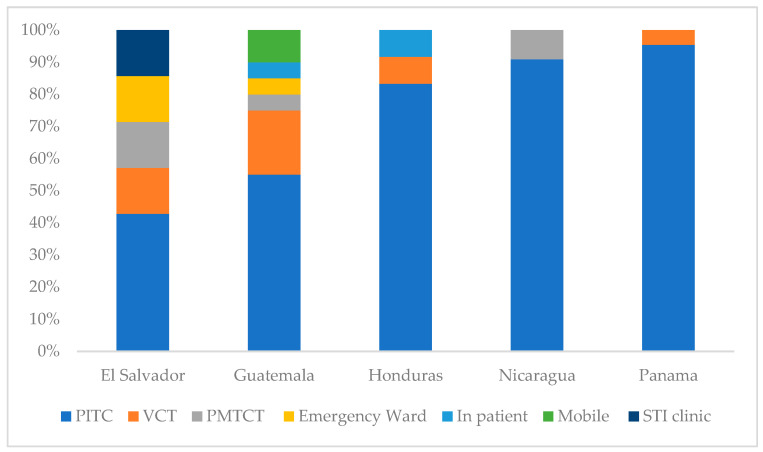
HIV positive test modality distribution by country from October 2023 to September 2024. Testing modalities: Emergency Ward = HIV testing in hospital emergency services; Inpat = HIV testing in inpatient hospital wards; PITC = provider-initiated testing and counseling during routine clinical visits; Mobile = community/mobile outreach testing services; PMTCT = prevention of mother-to-child transmission services; STI clinic = sexually transmitted infection; VCT = voluntary counseling and testing services.

- El Salvador consistently reported positivity below 1% throughout the period (Table 2) despite having the highest number of tests performed (5251), yielding 32 new diagnoses (0.6% positivity), all among those aged 15–19 year. Within the country, the highest percentages of positivity were, in descending order, Chalatenango, La Union, and La Paz (3.3%, 2.7%, and 1.6%, respectively).
- Guatemala had the second largest testing volume (4699), with its highest testing activity in the Guatemala department, whereas positivity was highest in Retalhuleu, Sacatepéquez, and Alta Verapaz (7.4%, 7.0%, and 5.6%, respectively). Chiquimula, which borders with Retalhuleu, had only 15 tests performed and 0% positivity, as did San Marcos.
- Honduras reported its highest proportion of tests conducted in Francisco Morazan (28%) and in Cortes (24%), with positivity being 1.3% and 1.7%, respectively; the highest positivity in Honduras was found in Colon (9.0%).
- Nicaragua observed the greatest variability over time, with positivity fluctuating from 7.0% in Year 1 to 1.4% in Year 4. The largest number of tests were performed in the capital, Managua, but positivity was only 1.7%. Atlantico Norte, with only 144 tests performed, had a positivity of 7.6%.
- Across all five countries, provider-initiated testing and counseling (PITC) accounted for the largest proportion of HIV-positive diagnoses, representing the predominant testing modality among adolescents with newly identified HIV infection (Figure 3).
- Panama conducted 2707 tests, with the highest number in its capital (1464 tests, 1.5% positivity). The highest positivity was recorded in Chiriqui (14.8%), followed by Bocas del Toro (9.7%) and Veraguas (5.3%); the Comarca Embera had no positive results (from 11 tests).

### 3.2. Treatment

#### 3.2.1. Adolescent Representation Within HIV Treatment Cohorts

Throughout the analysis period, an average of 1370 adolescents (10–19) were reported at year-end as being in PEPFAR-supported active HIV treatment cohorts in El Salvador, Guatemala, Honduras, Nicaragua, and Panama. This number reflects the average annual cohort size and does not represent unique individuals across the years. Regionally, these adolescents represent approximately 2.0% of the cohort in supported sites (Table 3, Supplemental Table S2).

Males represented 58% of all adolescents in the regional cohort. The highest proportions of male adolescents were observed in Guatemala (61%) and Panama (70%), whereas Honduras was the only country where females represented the majority (59%) of adolescents on treatment. The proportion of adolescents in the Honduras treatment cohorts have declined from 4.3% to 2.3% (1.9% decrease, *p* < 0.001).

In Year 4 (Table 3), adolescents represented 1.9% of the cohort in the Central American region, ranging from 1.2% in El Salvador to 2.7% in Nicaragua. Guatemala had the highest number of adolescents in FY24, representing 32% of all adolescents in the region.

#### 3.2.2. New to Treatment

**Figure 4:**
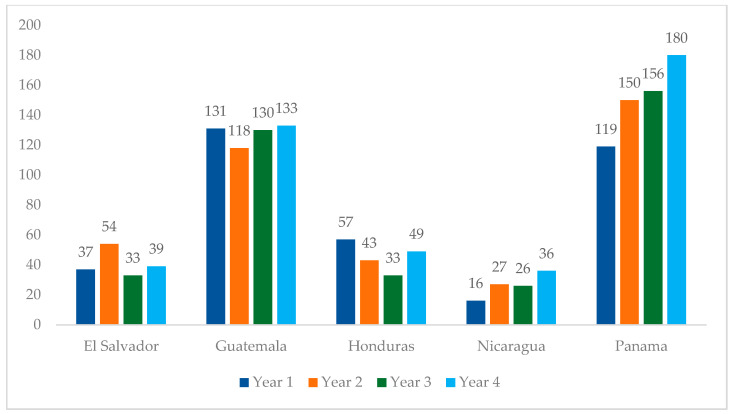
Regional overview of adolescent treatment initiations at PEPFAR-supported sites in Central America, October 2020–September 2024.

From Year 1 to Year 4 of this analysis, the number of adolescents initiating treatment (see Table 1 for indicator definitions) increased from 360 to 437, representing a 21% increase (Figure 4). This increase was mainly driven by Panama, which observed a 51% increase in treatment initiation during this period. Honduras was the only country in the region that reported fewer treatment initiations in Year 4 compared to Year 1, with a 14% decrease in treatment initiations. Nicaragua reported a 125% increase in this period. El Salvador and Guatemala observed marginal changes in treatment initiation from Year 1 to 4.

#### 3.2.3. Continuity of Treatment

Treatment interruption rates have remained relatively stable, fluctuating between 2% and 3% on average, and are generally lower and more stable than those observed in adults. Adult interruption peaked at 5.5% in Year 2, then gradually declined to 2.6% by the final quarter of Year 4. Nicaragua and Panama reported the highest average treatment interruptions, at 7.3% and 4.8%, respectively. The multi-month dispensing (MMD) of antiretroviral therapy (ART) increased, especially for 3–5-month refills, whereas 6-month dispensing remained low. Guatemala and Panama led in 3–5-month MMD periods, with each reporting over 50% MMD for adolescents in Year 4.

### 3.3. Viral Load Coverage and Suppression

**Figure 5:**
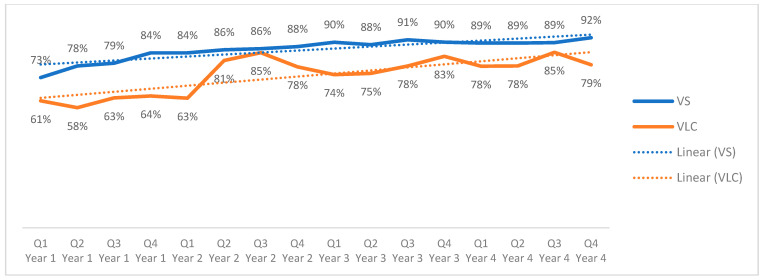
Regional viral load coverage and suppression in adolescents across quarters, October 2020–to September 2024.

Regional viral load coverage (Figure 5) showed a variable but overall upward trend among adolescents, increasing from 61% to 79% over the four-year period (an 18-percentage point increase, *p* > 0.10). At the regional level, viral load suppression among adolescents with a documented viral load result increased steadily from 73% in the first trimester of Year 1 to 92% in the last trimester of Year 4 (19-percentage point increase, *p* > 0.10). Because viral load suppression is calculated only among adolescents with a documented viral load result, these percentages reflect suppression among those with viral load testing and not among all adolescents on ART.

Viral load coverage and suppression demonstrated similar trends among adolescents aged 10–14 and 15–19 years. Viral load suppression among tested adolescents started below 90% in El Salvador, Guatemala, and Honduras in Year 1, but each reached 90% or above by the end of Year 4. The cohorts from Nicaragua reported a VLS of 86% in Year 1, which decreased to 81% by the end of the analyzed period. Panama reported the greatest improvement in VLS among those tested, with 60% virologic suppression reported in Year 1 and 86% reported in Year 4. Panama, although reporting an average of 56% coverage during this period, has seen significant improvements from 30% to 69% coverage. Although viral load coverage and suppression reflect changes across the analysis period, these results are not considered statistically significant with the available PEPFAR data (see Supplemental Table S3).

On average, viral load coverage among adolescents has varied across the region, with 74% reported regionally over the four-year period, indicating that roughly one quarter of adolescents on ART did not have a recent VL result recorded.

### 3.4. Advanced HIV

Data for advanced HIV disease (AHD) was only available for the final year of the analysis period given that this indicator was not included in previous PEPFAR guidance. Of adolescents new to treatment, 30% were categorized as having AHD. A difference between sexes (40% for women and 28% for men) was noted in the proportion of late-stage diagnoses; however, these differences were not statistically significant (see Supplemental Table S2). Panama and Guatemala have the highest percentages of AHD for both sexes (44% and 33%, respectively) and the highest percentage for women (44% and 53%, respectively). Honduras reported lower AHD among 15–19 years olds (10% in females; 15% in males). However, these values are likely underestimated, as the availability of CD4 results for adolescents in the region ranged from 8% missing in El Salvador to 56% missing in Panama, making age-specific comparisons difficult.

Overall, adolescent treatment outcomes showed modest improvements in initiation and continuity, whereas retention and viral load monitoring remain critical areas for strengthening across all programs.

## 4. Discussion

This study provides a multi-country descriptive analysis of adolescent HIV cascade outcomes across PEPFAR-supported programs in Central America, highlighting both progress and persistent gaps in testing, treatment, and viral suppression.

Although global estimates show that the burden of adolescent HIV is largely concentrated in sub-Saharan Africa (SSA) [14], the patterns observed in Central America remain relevant for understanding how small but concentrated epidemics respond to prevention and treatment intervention and how global averages can overshadow regional disparities [13,14,21].

### 4.1. Testing Patterns

Central America has maintained testing with a stable 2% positivity, unlike a previous analysis that documented declines in adolescent HIV testing attributed to service disruptions during COVID and shifts in PEPFAR guidance over similar time periods [22]. This could partly reflect how the involvement of regions with different epidemiological and programmatic contexts [5,23] can obscure trends in regions with smaller cohorts [13]. Despite the near parity in testing between males and females, the proportion of positives results was consistently higher in males. This pattern is consistent with the concentrated epidemic of the region [24,25] and differs from sub-Saharan Africa, where positivity is higher in females [14,26,27].

As we examined individual country patterns, both Guatemala and Panama maintained high HIV testing and positivity rates throughout the analyzed years, whereas Honduras also reports elevated positivity in specific populations. Beyond the context of a concentrated epidemic and the known vulnerability of adolescents to HIV [14,27], existing literature from these countries underscores the importance of considering indigenous populations such as the Ngäbe-Buglé, Garífuna, and Maya Q’eqchi’, who may also face vulnerabilities due to structural barriers, cultural and linguistic differences, and limited health services [13,28,29,30,31,32,33,34,35,36]. Although findings are sometimes inconsistent, these examples highlight both the importance of and the gaps in knowledge needed to tailor the HIV response to the realities of indigenous populations. Evidence from qualitative research suggests that age and ethnicity can act synergistically to deepen vulnerabilities to HIV. El Salvador had a 3% decrease in the incidence of HIV from 2000 to 2019 [37], which may reflect the cumulative impact of sustained national efforts to strengthen multiple prevention strategies [38], testing, and treatment within a primary health care framework, similar to decreasing trends reported in SSA [22]. Another indication of El Salvador’s epidemic control is the elimination of mother-to-child transmission of HIV, which may serve as a proxy indicator for the functionality of the country’s HIV program [39].

National HIV prevalence rates reported by UNAIDS in the analyzed countries range from 0.2 to 1.1 [40]. Positivity proportions among the adolescents included in this analysis ranged from 0.4% to 7.0%, which suggests some degree of targeted testing toward higher-risk adolescents. Although there is a predominance in provider-initiated and facility-based testing, such models may not fully align with adolescents’ preferences and needs [40,41,42]. In Sub-Saharan Africa, a systematic review of HIV uptake and yield among children and adolescents found that, although facility-based testing models reached higher-risk adolescents (late in disease progression) and therefore yielded more positive results, community- and family-based testing models facilitated earlier diagnosis [43]. Notably, index testing was not reported across the programs analyzed. This absence may reflect programmatic and ethical challenges in implementing contact testing among adolescents, similar to findings from a recent Ugandan study, which highlighted that less than one-third (28.7%) of adolescents living with HIV utilized provider-assisted partner notification, primarily due to potential violence and confidentiality concerns [44]. However, evidence from other high-burden settings demonstrates that index testing can achieve substantially higher yields than other testing modalities [45]. Peer- and network-based approaches, including social network strategy, leverage referrals within existing youth networks and have been associated with increased testing and case identification among young and high-risk populations [46] Because age shapes both risk and availability, strategies should differentiate between younger adolescents (10–14 years), more often reached though family/index testing and pediatric services, and older adolescents (15–19 years), who are more likely to seek services themselves and engage via peer/network approaches [47]. These findings underscore the need to expand adolescent-friendly outreach through strategies such as home visits, peer networks, family-based testing, self-testing, and potential adaptations of age-appropriate approaches to close remaining gaps in HIV detection among undiagnosed adolescents [42,48,49]. Additionally, restrictive adolescent consent laws in the region continue to limit the availability of HIV testing and prevention services, leaving healthcare providers uncertain about whether to prioritize the protection of minors or the promotion of self-care [50,51,52].

### 4.2. Treatment Pattern

Despite a 21% increase in new treatment initiation over the analyzed years, the overall proportion of adolescents within the HIV treatment cohorts remained stable. This suggest that although linkage to treatment has improved in some countries such as Panama and Nicaragua, many new initiations may have been balanced out by the transition to adult services. The consistently small cohorts in El Salvador and Panama could also reflect challenges in early diagnosis and long-term retention.

This pattern of increased ART initiation without a proportional increase in the cohort is not unique to Central America. In Lima, Peru, adolescents and young adults experienced low retention despite initiating ART, a trend largely attributed to structural barriers such as clinic hours, mental health morbidity, and the ineffective transition to adult care. Many adolescents’ initiating treatment between the ages of 15 and 19 were enrolled directly into adult services [53]. The age of initiation and lack of transitioning services in this age group should be considered in Central America.

Treatment continuity trends revealed decreasing interruption rates and an expansion of multi-month dispensing of ART. By year 4, interruption rates among adolescents and adults converged, suggesting some narrowing of the gap in program continuity. However, these improvements may still be fragile, and we were not able to calculate individual-level retention with aggregate MER data. The higher proportion of males on treatment (58%) may also highlight the cumulative effect of testing patterns, where adolescent males, although less likely to test, were more likely to test positive and thus appear in treatment cohorts. This pattern emphasizes the need for differentiated care models that address the needs of both sexes. Sustained retention among adolescents requires multiple layered approaches, including community and virtual clinical services, psychosocial support, peer support, and trained providers to address age-specific needs [48]. Although retention issues in our region remain modest, a combination of interventions could stabilize and progressively strengthen retention over time, particularly among male adolescents.

### 4.3. Viral Load Coverage and Suppression Patterns

Adolescents persistently lag behind adults in virologic suppression worldwide because of their unique barriers in adhering to treatment: developing executive function, lack of systemic support via school-based programs, and psychosocial challenges that require adolescent-specific treatment strategies and specialized support approaches beyond those traditionally offered for adults [54,55]. In our analysis, virologic suppression among those with a viral load result improved over time; however, viral load coverage remained incomplete, meaning that a substantial proportion of adolescents on ART did not receive a documented viral load test. Because suppression is typically calculated only among those tested, viral load results may overestimate true population-level suppression when coverage is suboptimal. Therefore, interpreting adolescent viral load outcomes requires considering both coverage and suppression together, rather than either indicator alone. Continued efforts to scale up viral-load testing and coverage remain essential to achieve more complete monitoring of adolescent treatment outcomes and to support the accurate assessment of program progress [56].

In Panama, where adolescents exhibited high positivity but smaller treatment cohorts, drug resistance may complicate viral suppression outcomes. A recent study by Ventosa-Cubillo et al. (2023) found that more than half of children and adolescents living with HIV carried major drug resistance mutations, primarily to NRTIs and NNRTIs [57] Some ART-naïve adolescents were already infected with resistant strains, indicating the ongoing transmission of resistant viruses. These findings underscore the need for routine surveillance and robust adherence support, highlighting that improvements in viral load suppression will not only depend on programmatic adherence but also on the availability of effective regimens [58].

Beyond pharmacologic monitoring, stronger adolescent support is still essential. Regional efforts to increase peer support programs and leverage adolescent-friendly mobile health technologies are needed to improve adolescent virologic suppression. Similarly, differentiated service delivery models shown to support adolescent adherence and retention should be implemented throughout the region, including MMD for adolescents and adolescent friendly clinics [1]. Family dynamics, school schedules, transportation limitations, and developmental needs should all be factored into “adolescent-friendly” services [59,60].

### 4.4. Advanced HIV Disease at Diagnosis

The high prevalence of AHD and the fact that PITC is the highest-yield testing modality among adolescents new to treatment (34%) highlight missed opportunities for early detection. Late presentation and advanced HIV disease has already been documented in Latin America [61]. The sex difference (40% females and 28% males) suggest that women may be facing additional barriers, contrary to a study that found that sex was a significant predictor of advanced HIV disease (AHD) among young people in Sierra Leone, with females demonstrating significantly lower odds of having AHD compared to males [62]. As noted above, most testing takes place at health facilities, whereas current research suggests that adolescents prefer more community-based or home-based testing [1]. For epidemics such as Central America’s, which are concentrated primarily among high-risk populations, integrating testing and prevention into community spaces may be a future focus to better address issues related to privacy and confidentiality concerns. Integral to testing programs in community are focused resources to link adolescents who test positive for HIV to care. Without this crucial step, AHD rates will remain elevated. Higher rates of AHD among women may reveal gaps in community-based testing for young women or structural barriers to prevention, testing, and linkage to care among adolescent young women. Efforts to screen for and address violence may be a crucial component of HIV testing outreach for adolescent women [62,63].

It is important to highlight that the interpretation of advanced HIV disease among adolescents requires caution because data were only available for the final year. Similar to VL documentation, data completeness varies substantially across countries, which could lead to the underestimation or overestimation of values.

### 4.5. Limitations and Strengths

This analysis is subject to several limitations:

- It relies on routine programmatic data from DATIM, which are de-identified, aggregated, and not patient-level, limiting the ability to explore individual outcomes across the HIV cascade or evaluate person-level retention or adherence. This introduces the possibility that aggregated numbers may not represent individual data.
- Given the aggregate nature of these data, the timing of infection remains unclear in these adolescents. If these are missed perinatal transmissions, regulations should be focused on improved testing for infants and toddlers with HIV exposure. If these are primarily new infections from sexual activity, guidelines should focus on HIV prevention among adolescents. Further, patient-level data is needed to help guide these initiatives.
- Viral load suppression is reported only among those with a documented viral load result, whereas viral load coverage was incomplete, varying across countries and years. This could lead to an overestimation of true population-level suppression.
- Data completeness varies across countries, particularly for CD4 testing, which is essential to determine AHD status. CD4 results were available only for the final year and missing data limits our ability to assess AHD reliably or make comparisons across sexes or age groups.
- Key disaggregations by high-risk populations, migration status, and ethnicity were not available, restricting further analysis of vulnerable populations. Other indicators associated with the adolescent HIV cascade, such as behavioral, socioeconomic, or structural barriers, are not included in routine MER reporting, which may limit interpretation.
- Finally, because the analysis includes only PEPFAR-supported sites, findings may not represent all adolescents receiving HIV services nationally.

Despite these limitations, the study’s strengths include its multi-country scope, standardized MER definitions, and the use of core cascade indicators to provide a regional perspective for adolescents in Central America, which is rarely available in our region.

## 5. Conclusions

Across Central America, adolescent HIV outcomes showed areas of progress alongside persistent programmatic gaps. Testing volume was maintained over the four-year period, yet case detection remained concentrated among older adolescents and males, and a substantial proportion of new diagnoses presented with advanced HIV disease. Although viral load suppression among those tested improved, incomplete viral load coverage may indicate that many adolescents on ART are not yet fully captured in monitoring systems. These patterns underscore that further gains in adolescent outcomes will depend on continued attention to earlier diagnosis, consistent viral load monitoring, and age-responsive service delivery. The analysis also highlights the diversity of testing modalities and treatment cohorts across countries, shaped by differences in service availability, reporting practices, and epidemic profiles. Approaches such as confidential, age-appropriate outreach, social and peer-network strategies, re-evaluation of the age of testing consent laws, and adaptations of partner-based testing—already used in other settings—illustrate potential avenues through which programs in the region can expand reach and support adolescent engagement.

Although this study was not designed to evaluate specific interventions, the findings point to opportunities for countries to draw on existing national platforms and experience to strengthen adolescent-focused components of their HIV programs as they pursue progress toward the 95-95-95 goals.

## Figures and Tables

**Table 1:** Definitions of Core PEPFAR Monitoring, Evaluation, and Reporting (MER) indicators for HIV for the reporting periods.

MER Indicator	Indicator Description	Definition
HTS_TST	HIV testing	The number of individuals who received HIV testing services and received their results
HTS_TST_POS	HIV-positive test	Number of individuals who received HIV testing services and were diagnosed HIV-positive
TX_NEW	New treatment initiations	The number of individuals newly enrolled on antiretroviral therapy
TX_CURR	Currently on treatment	The number of individuals receiving antiretroviral therapy at the end of the reporting period
TX_ML	Treatment interruption	The number of people who were on ART but then interrupted treatment during the reporting period
TX_PVLC	Viral load coverage	Number of ART patients with a viral load test result documented within the past 12 months
TX_PVLS	Viral load suppression	Percentage of ART patients with a suppressed viral load (<1000 copies/mL) documented within the past 12 months.
MMD	Multi-month dispensing	Number of ART patients who received ≥3 months of ART dispensed at their last pick-up

Taken from the Monitoring, Evaluation, and Reporting Reference Guide [17], Waldrop [20].

**Table 2:** HIV testing outcomes in Central America from October 2020 to September 2024.

	# HIV Tests					# HIV-Positive Tests				
Year	I	II	III	IV	Total	I	II	III	IV	Total
Age										
10–14	958	1485	1312	1694	5449	13	15	11	12	51
15–19	10,457	10,967	14,246	15,543	51,228	221	298	347	326	1192
Sex										
Female	7052	5432	6083	7374	25,943	33	62	84	62	241
Male	4363	7020	9475	9863	30,734	201	251	274	276	1002
Country										
El Salvador	2213	3172	2979	5251	13,615	14	30	21	32	97
Guatemala	6276	3295	3767	4699	18,037	97	107	125	122	451
Honduras	1846	2859	2625	2688	10,018	54	51	38	46	189
Nicaragua	330	530	3250	1892	6002	23	29	45	35	132
Panama	750	2596	2937	2707	8990	46	96	129	103	374
Total	11,415	12,452	15,558	17,237	56,662	234	313	358	338	1243

**Table 3:** Distribution of adolescents on treatment in comparison with the general treatment cohort in PEPFAR supported sites from October 2023 to September 2024.

Country	# of Adolescents	Total Cohort	% of Adolescents
El Salvador	184	15,551	1.2%
Guatemala	440	19,394	2.3%
Honduras	285	12,229	2.3%
Nicaragua	147	5520	2.7%
Panama	305	18,289	1.7%
Regional	1361	70,983	1.9%

Note: Data for Years 1–3 of ALHIV on treatment, in comparison with the PEPFAR cohort, can be found in Supplemental Table S4.

## Data Availability

The data analyzed in this study were obtained from the U.S. President’s Emergency Plan for AIDS Relief (PEPFAR) Data for Accountability, Transparency, and Impact Monitoring (DATIM) system. Access to DATIM data is restricted to authorized users and implementing partners. Therefore, the data is not publicly available due to programmatic confidentiality agreements. Aggregated data supporting the findings of this study may be available from the corresponding author upon reasonable request and with permission from PEPFAR.

## Supplementary Materials

The following supporting information can be downloaded at: https://www.mdpi.com/article/10.3390/tropicalmed11010005/s1, Supplemental Table S1: HIV testing and treatment outcomes in Central America, October 2020 to September 2024; Supplemental Table S2: Comparison of HIV positivity yield, advanced HIV disease results, and Honduras adolescent and adult cohorts, by sex, Supplemental Table S3: Viral load coverage and suppression proportions by country, Year 1 vs. Year 4, Supplemental Table S4: Adolescent and total cohort size by country, October 2020 to September 2024, Supplemental Figure S1: HIV testing among adolescents in Central American countries supported by PEPFAR, October 2020 to September 2024, Supplemental Figure S2: Positive HIV tests among adolescents in Central American countries supported by PEPFAR, October 2020 to September 2024, Supplemental Figure S3: Adolescent users new to treatment in Central American countries supported by PEPFAR, October 2020 to September 2024, Supplemental Figure S4: Total adolescents on treatment in Central American countries supported by PEPFAR, October 2020 to September 2024, Supplemental Figure S5: Treatment interruptions among adolescents in Central American countries supported by PEPFAR, October 2020 to September 2024.

## Abbreviations

The following abbreviations are used in this manuscript:

AHD	Advanced HIV Disease
ART	Antiretroviral Therapy
CDC	U.S. Centers for Disease Control and Prevention
CD4	Cluster of Differentiation 4 (T lymphocyte count)
ELS	El Salvador
FY	Fiscal Year
GUA	Guatemala
HIV	Human Immunodeficiency Virus
HON	Honduras
HTS	HIV Testing Services
MMD	Multi-Month Dispensing (of ART)
MER	Monitoring, Evaluation, and Reporting (PEPFAR reporting framework)
NIC	Nicaragua
PAN	Panama
PEPFAR	U.S. President’s Emergency Plan for AIDS Relief
PITC	Provider-Initiated Testing and Counselling
PMTCT	Prevention of Mother-to-Child Transmission
SSA	Sub-Saharan Africa
STI	Sexually Transmitted Infection
UNAIDS	Joint United Nations Programme on HIV/AIDS
VCT	Voluntary Counselling and Testing
VLC	Viral Load Coverage
VLS	Viral Load Suppression
WHO	World Health Organization
