# Supplementary material for: Analysis of Adolescent HIV Care Cascade Outcomes in PEPFAR-Supported Programs in Central America, October 2020–September 2024
Source: Trop Med Infect Dis. 2025 Dec 24;11(1):5. doi: 10.3390/tropicalmed11010005 (PMC12846695; doi:10.3390/tropicalmed11010005)

Supplemental Table S1: HIV testing and treatment outcomes in Central America, October 2020 to September 2024

Number of HIV Tests	Year 1	Year 2	Year 3	Year 4	p-value*
**Age**					
10-14	958	1485	1312	1694	<0.001
15-19	10457	10967	14246	15543	
**Sex**					
Female	7052	5432	6083	7374	<0.001
Male	4363	7020	9475	9863	
**Country**					
El Salvador	2213	3172	2979	5251	<0.001
Guatemala	6276	3295	3767	4699	
Honduras	1846	2859	2625	2688	
Nicaragua	330	530	3250	1892	
Panama	750	2596	2937	2707	
**Number of Positive Test**					
**Age**					
10-14	13	15	11	12	<0.001
15-19	221	298	347	326	
**Sex**					
Female	33	62	84	62	<0.001
Male	201	251	274	276	
**Country**					
El Salvador	14	30	21	32	<0.001
Guatemala	97	107	125	122	
Honduras	54	51	38	46	
Nicaragua	23	29	45	35	
Panama	46	96	129	103	
**New to treatment**					
El Salvador	37	54	33	39	0.009
Guatemala	131	118	130	133	
Honduras	57	43	33	49	
Nicaragua	16	27	26	36	
Panama	119	150	156	180	

Treatment interruptions					
El Salvador	23	33	22	12	<0.001
Guatemala	50	45	36	42	
Honduras	87	47	48	22	
Nicaragua	16	30	23	7	
Panama	23	107	65	51	
**Total Adolescents on Treatment**					
El Salvador	256	228	197	184	<0.001
Guatemala	484	468	477	440	
Honduras	437	313	286	285	
Nicaragua	57	68	102	147	
Panama	167	312	265	305	

*Chi-squared testing was employed to estimate p-values

Supplemental Table S2: Comparison of HIV positivity yield, advanced HIV disease results, and Honduras adolescent and adult cohorts, by sex

	Males	Females	p-value
**Positivity Yield**			
10-14	1.60%	0.20%	Male: p>.10**
15-19	2.90%	1.00%	Female: p>0.10**
**Advanced HIV Disease**			
CD4<200	67	22	0.52*
CD4>=200	273	75	
**Honduras Adolescent Cohort**			<0.001*
10-19	437	285	
All Ages	10331	12229	

*Chi-squared testing was employed to estimate p-value

**Two-sample proportion testing was employed to estimate p-values

Supplemental Table S3: Viral load coverage and suppression proportions by country, Year 1 vs. Year 4

Viral load coverage	Year 1	Year 4	p-value**
El Salvador	90%	90%	p>0.10
Guatemala	80%	80%	p>0.10
Honduras	73%	85%	p>0.10

Nicaragua	86%	95%	p>0.10
Panama	30%	69%	p>0.10
Regional	61%	80%	p>0.10
**Viral load suppression**			
El Salvador	88%	94%	p>0.10
Guatemala	86%	91%	p>0.10
Honduras	77%	90%	p>0.10
Nicaragua	86%	81%	p>0.10
Panama	60%	86%	p>0.10
Regional	79%	90%	p>0.10

**Two-sample proportion testing was employed to estimate p-values

Supplemental Table S4: Adolescent and total cohort size by country, October 2020 to September 2024

	Year 1			Year 2			Year 3			Year 4		
**Country**	**# of Adolescents**	**Total Cohort**	**% of Adolescents**	**# of Adolescents**	**Total Cohort**	**% of Adolescents**	**# of Adolescents**	**Total Cohort**	**% of Adolescents**	**# of Adolescents**	**Total Cohort**	**% of Adolescents**
El Salvador	256	13,195	1.9%	228	14,430	1.6%	197	14,945	1.3%	184	15,551	1.2%
Guatemala	484	21,238	2.3%	468	22,299	2.1%	477	23,492	2.0%	440	19,394	2.3%
Honduras	437	10,331	4.2%	313	10,608	3.0%	286	11,085	2.6%	285	12,229	2.3%
Nicaragua	57	1,485	3.8%	68	3,294	2.1%	102	4,810	2.1%	147	5,520	2.7%
Panama	167	12,846	1.3%	312	16,264	1.9%	265	17,530	1.5%	305	18,289	1.7%
Regional	1401	59,095	2.4%	1389	66,895	2.1%	1327	71,862	1.8%	1361	70,983	1.9%

Supplemental Figure S1: HIV testing among adolescents in Central American countries supported by PEPFAR, October 2020 to September 2024

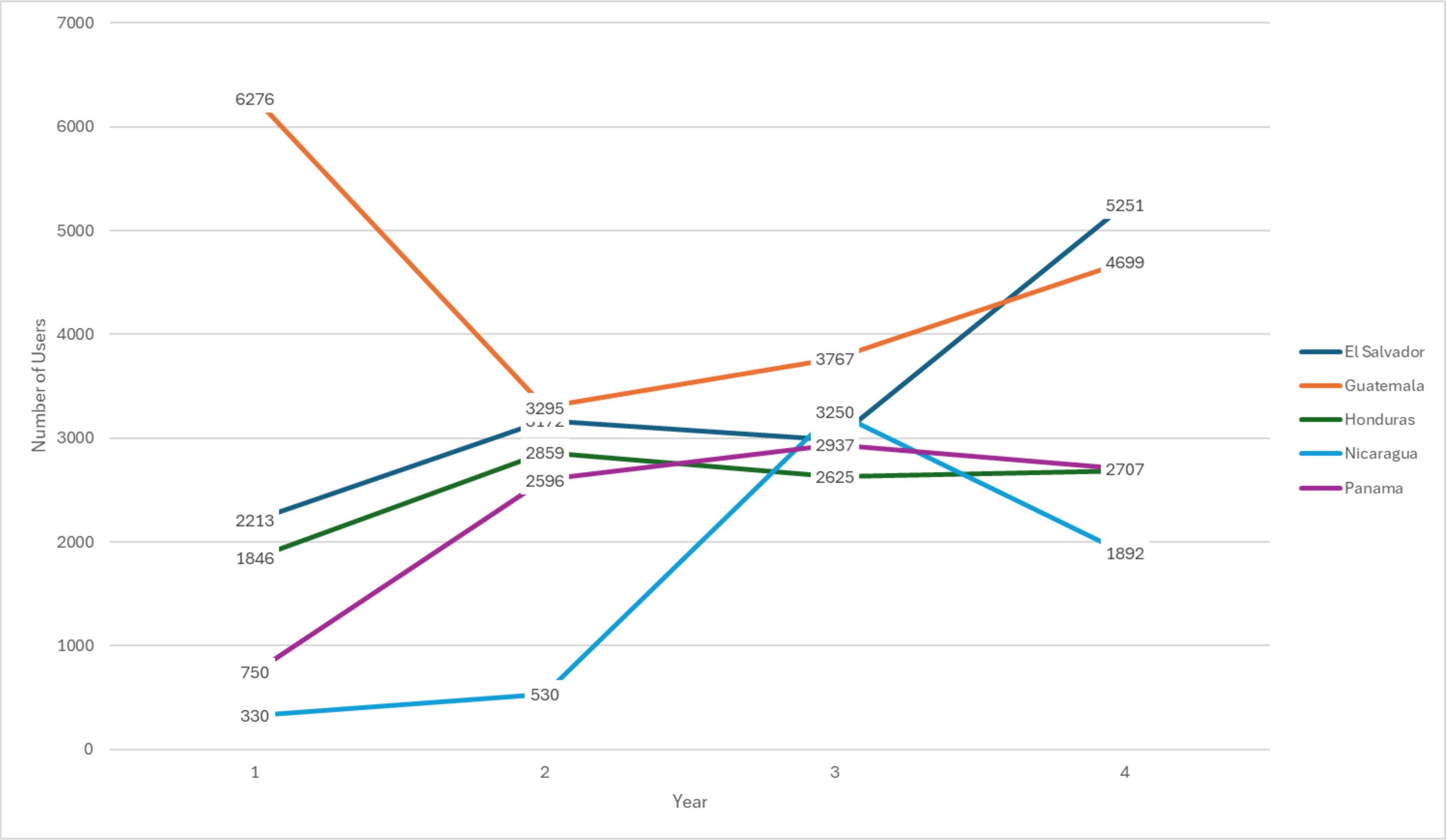

Supplemental Figure S2: Positive HIV tests among adolescents in Central American countries supported by PEPFAR, October 2020 to September 2024

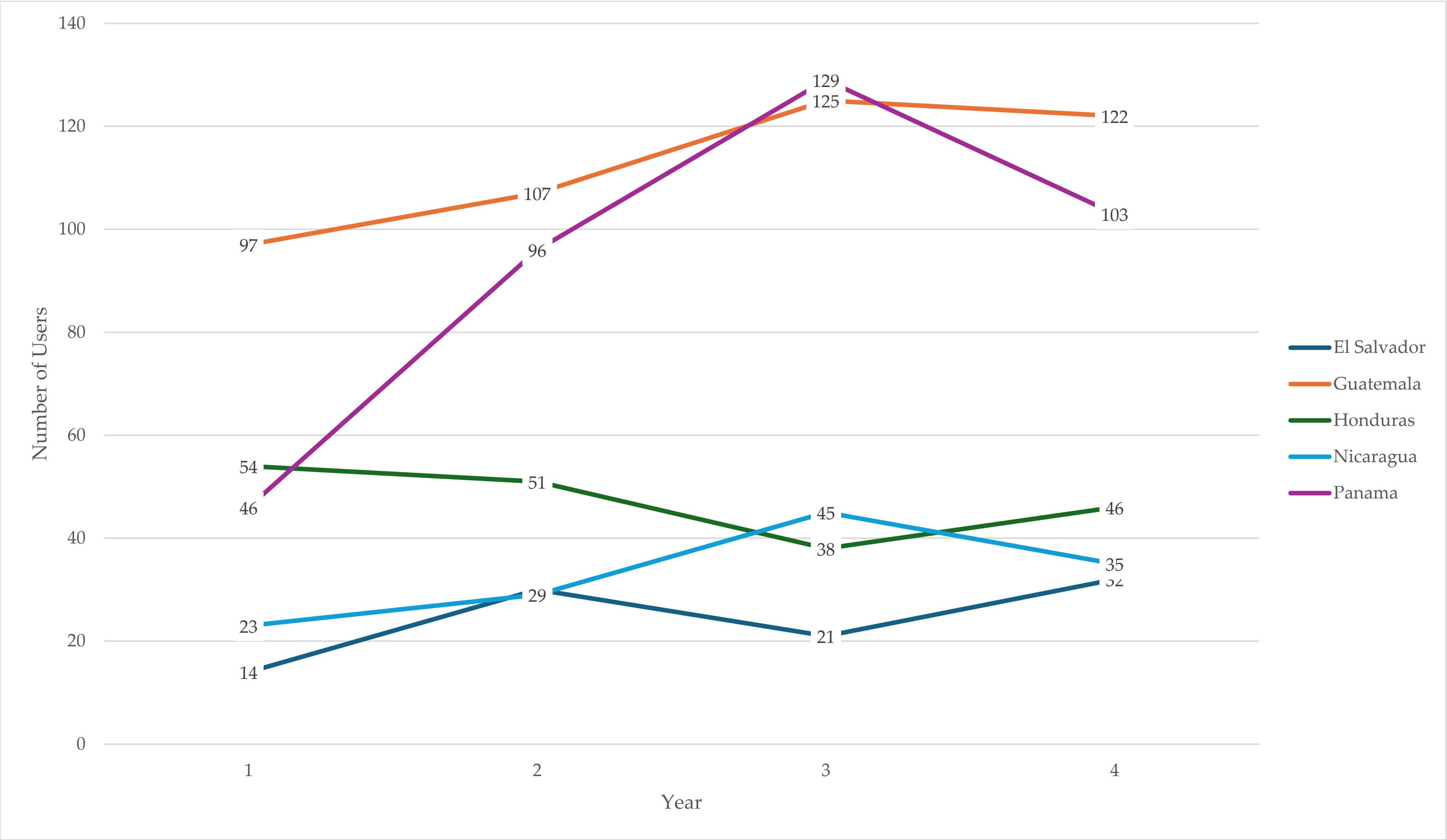

Supplemental Figure S3: Adolescent users new to treatment in Central American countries supported by PEPFAR, October 2020 to September 2024

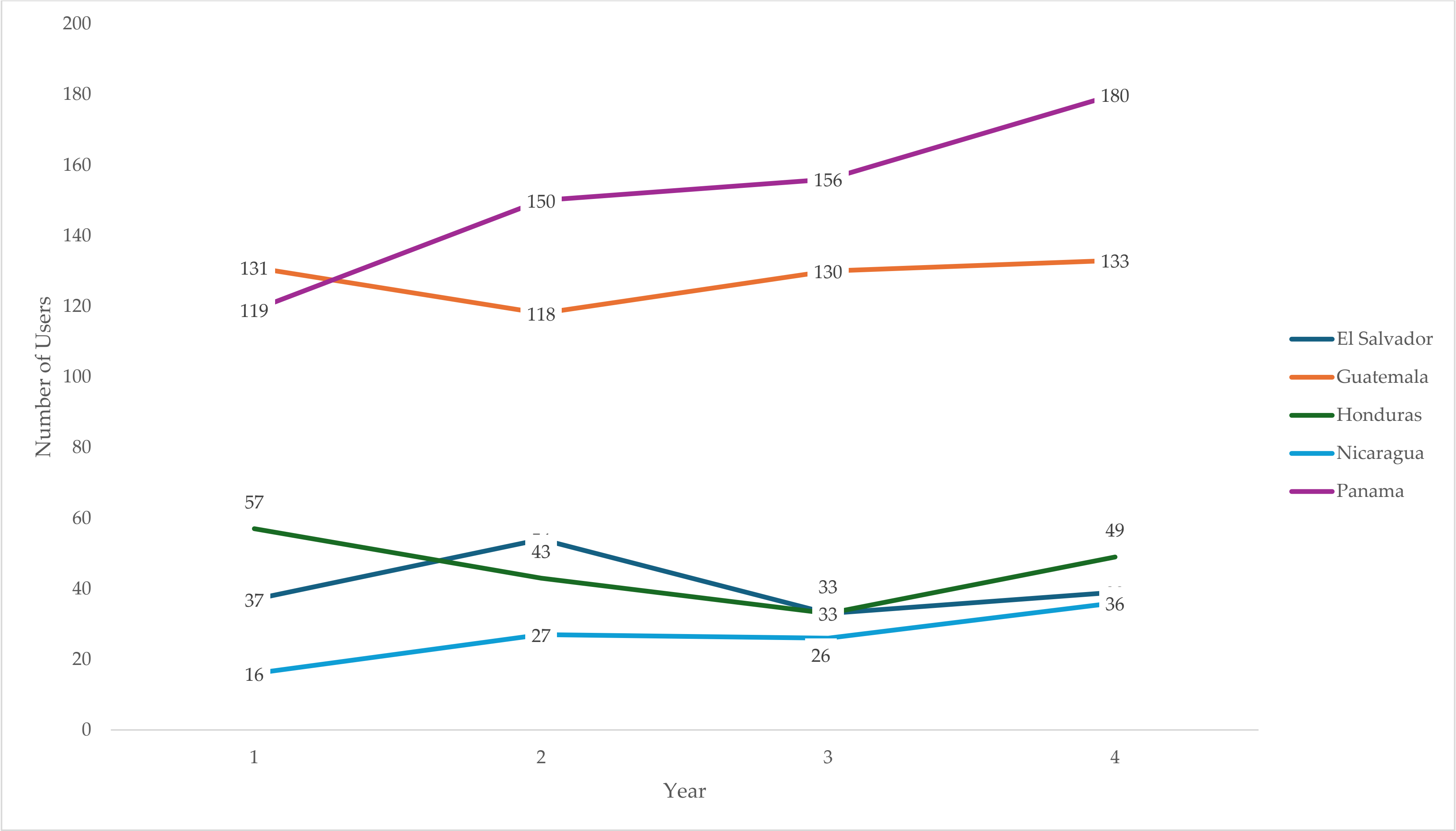

Supplemental Figure S4: Total adolescents on treatment in Central American countries supported by PEPFAR, October 2020 to September 2024

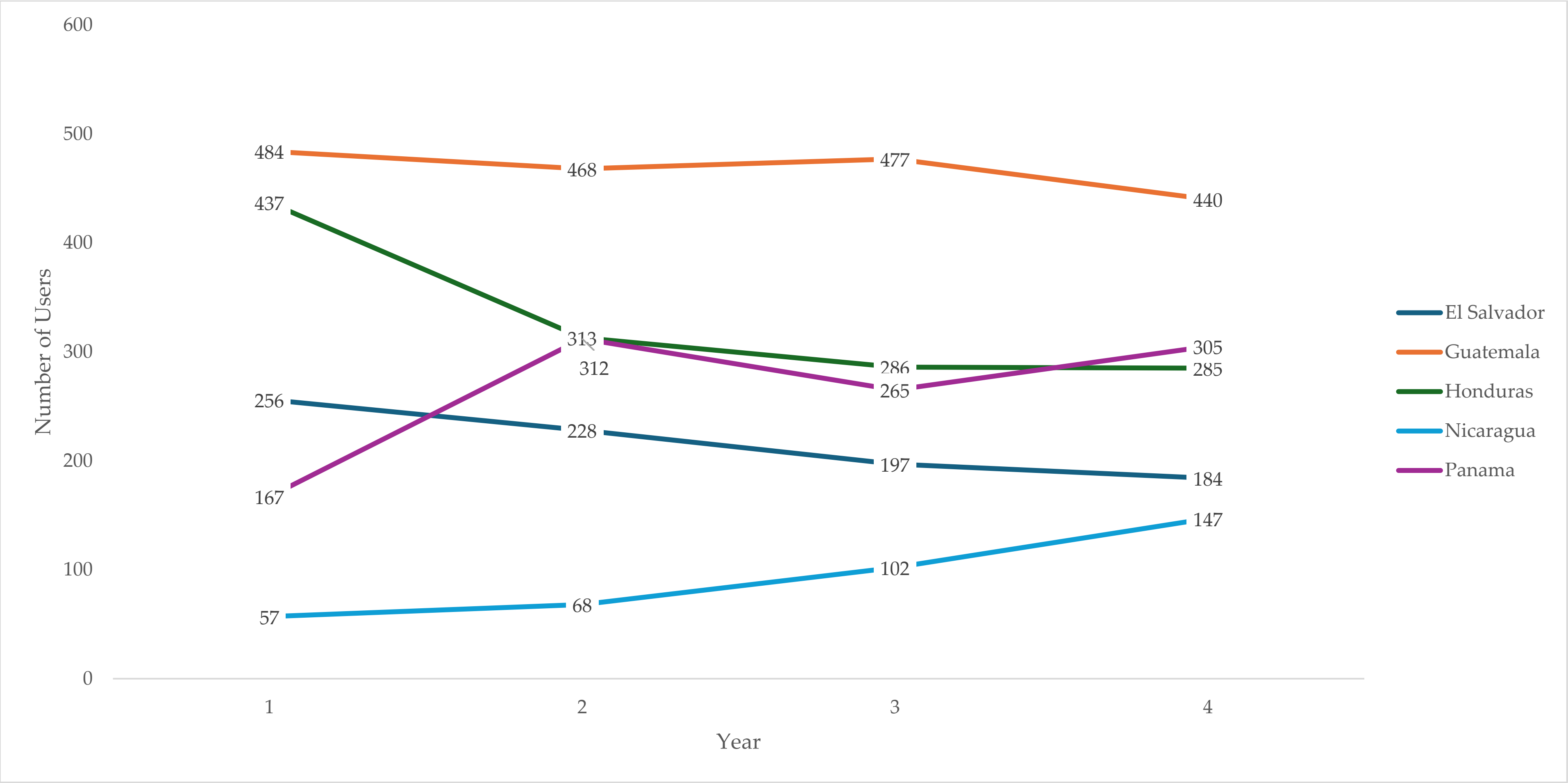

Supplemental Figure S5: Treatment interruptions among adolescents in Central American countries supported by PEPFAR, October 2020 to September 2024

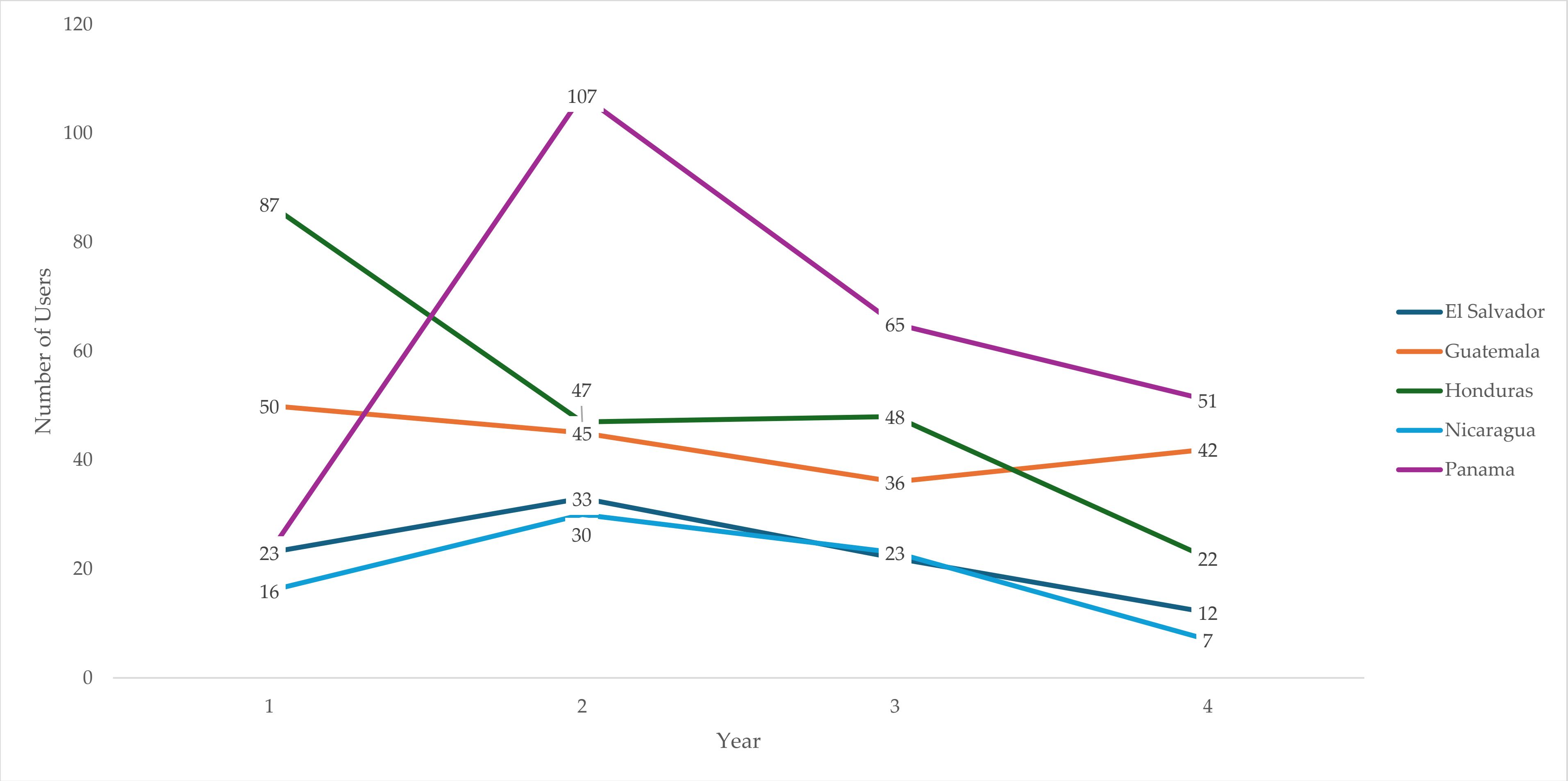

Supplement: Supplementary file 1 [file tropicalmed-11-00005-s001.zip › tropicalmed-3988950-supplementary.pdf]
